# Evaluation of quality of life and associated factors among HIV patients on antiretroviral therapy in North West region of Cameroon

**DOI:** 10.4314/ahs.v21i1.3S

**Published:** 2021-05

**Authors:** Anissette N Busi, Marius Nsoh, Moses O Otieno, Sylvester A Ndeso, Gregory E Halle-Ekane

**Affiliations:** 1 Georgetown University Center for Global Health Practice and Impact; TIDE Project, Cameroon; 2 Department of Public Health, School of Health Sciences, Catholic University of Central Africa; Cameroon; 3 National AIDS and Sexually Transmitted Infections Control Program (NASCOP); Kenya; 4 Department of Public Health, Faculty of Health Sciences, University of Buea; Cameroon

**Keywords:** Quality of life and associated factors, HIV patients, antiretroviral therapy, Cameroon

## Abstract

**Background:**

There is evidence that Quality of Life (QoL) of People Living with HIV/AIDS (PLHIV) has a significant role in ART retention, treatment adherence, and survival. As a result, QoL is becoming increasingly important for policymakers, program implementers, and researchers. However, factors associated with QoL, in a culturally diverse country like Cameroon are unknown.

**Objective:**

We aimed to assess the QoL of PLHIV on ART and assess the extent to which physical, psychosocial, environmental, and spiritual factors drive QoL.

**Method:**

A cross-sectional study was conducted among 394 PLHIV aged >21 in North-West Cameroon from April to July 2019. Data were collected using WHO-QOL BREF questionnaire. Descriptive statistics, bivariate, and multivariate linear regression analyses were performed.

**Results:**

Majority (34.5%) of participants were in the age range of 41–50, with 73% females. The average QoL of the respondents was “good” with mean score of 3.57 on 5 and 71.4% agreed to have satisfactory QoL. Bivariate regression analyses revealed that all six proposed predictors were significantly associated with QoL. Psychological factors made the greatest impact (β = 0.213; p<0.003), followed by physical factors (β = 0.19; p<0.001).

**Conclusion:**

PLHIV fairly agreed to have good QoL. The QoL was driven by mainly psychological and physical factors and not level of independence. However, the mean score perceptions for the investigated domains were low. Mental health services should consider these predictors when designing strategies to improve the QoL of PLHIV. While this study provides useful insights, other possible drivers of QoL among PLHIV should be investigated.

## Introduction

The World Health Organization has defined QoL as “individuals' perceptions of their position in life in the context of the culture and value systems in which they live and in relation to their goals, standards, expectations, and concerns” 1. This definition reflects the view that QoL refers to a subjective evaluation that is embedded in a cultural, social, and environmental context. Because this definition of QoL focuses upon respondents' “perceived” QoL, it is not expected to provide a means of measuring in any detailed fashion symptoms, diseases or conditions, but rather the effects of disease and health interventions on QoL. As such, QoL cannot be equated simply with the terms “health status”, “lifestyle”, “life satisfaction”, “mental state” or “well-being”. 1

Globally, there are about 33 million HIV infected people. Sub-Saharan Africa alone accounts for 67% of these infected people although only 13% of the world's population reside in Sub Saharan Africa 2,3. As of 2017, Cameroon has an adult HIV prevalence of 3.7 % (3.0–4.3) down from a prevalence of 4.4 in 2015 among adults aged 15 to 49 years with women having twice the prevalence of men (women 4.8; men 2.5) 2, 3.

HIV is being considered a chronic disease. For PLHIV, this means having to cope with a range of HIV-related symptoms for extended periods. Symptoms may be related to the infection itself, comorbidities, or iatrogenic effects from HIV-related medications 4,5. Many of the HIV patients struggle with numerous social problems such as stigma, poverty, low self-esteem, depression, substance abuse, and cultural beliefs which can affect their QoL not only from the physical health aspect but also from a mental and social health point of view and cause numerous problems in useful activities and interests of the patients 6.

Antiretroviral Therapy (ART) can improve survival, reduce the occurrence of HIV-related opportunistic infections, and improve the patients' overall wellbeing 7. With this innovation in the lives of HIV patients, it becomes important to holistically assess the health of such patients in view of their social, psychological, spiritual, and environmental wellbeing as these can significantly affect the effectiveness of the drugs they receive 8,9. For example, studies show that stressful events and poor social support were related to HIV-1 disease progression to AIDS. In addition, research on the psychosocial aspects of HIV- positive status reveals that being HIV positive is associated with a large measure of stress and depression 10,11. Therefore, the significance of this study is two folds. First, assessing the psychosocial, spiritual, and environmental state of clients on ART will provide feedbacks on non-clinical factors that can significantly affect the given treatment. On the other hand, findings on clients' perception on their health-related QoL while on ART will inform health care providers and other key players in the care and treatment of HIV/AIDS on the effectiveness of the therapy they provide, and the compliance of the patients on prescribed regimens and counseling services provided. This will contribute to overall service improvement in HIV/AIDS treatment cascade through the designing of interventions that address the factors most strongly associated with poor QoL among patients on ART. This study has as objectives to;

- Assess clients' perception of their Quality of Life while on ART
- Assess clients' perception of their physical and psychological wellbeing while on ART in NW Cameroon
- Assess clients' perception of their social support and relationships while on ART in NW Cameroon.
- Assess clients' perception of their environmental status while on ART in NW Cameroon.
- Assess clients' perception of their Spirituality/Religious/Personal Beliefs ( SRPB) while on ART in NW Cameroon

This study seeks to assess the QoL among patients on ART in two high volume sites in North West Cameroon to ascertain the effectiveness of the provided care because a patient on ART who is virally suppressed but cannot return to his job or other social affiliations for fear of stigmatization or remains psychologically depressed is still “sick” from a holistic point of view. This study is in line with the WHO definition of health which states that “Health is a state of complete physical, mental and social well-being and not merely the absence of disease or infirmity” 12.

## Methods

### Study setting and study design

An institution-based cross-sectional study conducted among PLHIV on ART from two high volume sites in the North West Region of Cameroon namely; Nkwen Baptist Health Centre and Mbingo Baptist Hospital which are both Faith-based institutions. Participants of the study were enrolled and interviewed during their routine ART pick-up appointments at the health facility. The treatment units had 3629 and 1457 active clients on ARV respectively as of December 31^st^ 2018. In these centers, ARVs are dispensed monthly or three months based on the availability of the drugs, client's distance from the point of collection, client's duration, and adherence to treatment and client's clinical state. The following services are available at the treatment units;

- Nutrition counselling which is done by a nutrition counsellor
- Psychosocial/psychospiritual support
  - Spiritual counselling is done by chaplains
  - Adherence counselling is done by social workers, Psychosocial agents (APS) and nurses
- Clinical HIV care is provided by the site physicians and senior nurses trained in HIV care
- Pharmacy services
- Laboratory investigations and monitoring services with HIV testing, viral load testing, CD4 etc.
- TB screening and treatment services.

The majority (90%) of the study population expressed themselves conveniently in English or Pidgin English while the other 10% understood either only French or the “Kom” Language.

### Data Collection method and tool

Data were collected using a validated structured questionnaire which comprises WHO QoL HIV shortform instrument (WHOQOL-HIV BREF) items. The WHOQOL-HIV BREF contains 31 items distributed into 6 domains: physical, social relationships, level of independence, and spirituality domains each had 4 items. Psychological and environmental domains had 5 and 8 items, respectively. The individual items are rated on a 5-point Likert scale where 1 indicates low/negative perceptions and 5 indicates high/positive perceptios. The remaining two items measured overall perceived QoL and general health perception of PLHIV on ART 1.

The questionnaire was administered by the Principal Investigator (PI) with the help of the Heads of the care and treatment units. For those who were not literate, the PI interpreted verbally in Pidgin English the information sheet, consent form, and questionnaire according to the reading and understanding level of the respondents to enable comprehension and the participants were given time to answer each question. Those who could not understand English nor Pidgin English, or who were less than 21 years were excluded from the study.

### Sample size

The sample size was calculated using Lorenz equation. With a confidence level of 95% (i.e. z=1.96) an error margin of 5%, a sample of 358 participants was computed using the equation below

$$\mathrm{Sample}\;\mathrm{Size}=\frac{\frac{z^{2}\times p(1-p)}{e^{2}}}{1+(\frac{z^{2}\times p(1-p)}{Ne^{2}})}$$

Where N= population size, e=margin of error and z=z score, p= probability of success set at 50%

The total number of client's on treatment at these 2 units was 5086. To improve the accuracy of the study, a 10% non-response rate was added to give a total sample of 394. Then proportionate sampling was done to get the proportionate samples from the two sites as follows Sample from Nkwen Baptist Health Center=

$$\frac{3629\times 394}{5086}\begin{array}{c} =\text{281} \end{array}$$

Sample from Mbingo Baptist Hospital=$\frac{1457\times 394}{5086}\begin{array}{c} =\text{113} \end{array}$

### Data Cleaning and Analysis

To ensure that the data was correctly collected, descriptive statistics were done to identify missing data. Raw scores of each item were also determined to identify outliers. No missing data nor outliers were found.

Before the data was analyzed, Normality test was done using Pearson test at confidence interval of 95% indicating a normally distributed dataset. Then negative questions were recoded as stipulated by the manual for WHOQOL-HIV. With guidance from the WHO manual, items were grouped under their respective domains and Composite scores of the main variables/domains (general, physical, psychological, social relations, environmental, and SRBP) were obtained. A two-step regression analysis was done. The first step was a bivariate analysis between the dependent variable and each of the five independent variables. This was followed by a multivariable between the dependent variable and the independent variables.

## Results

### Demographics

**Figure 1:**
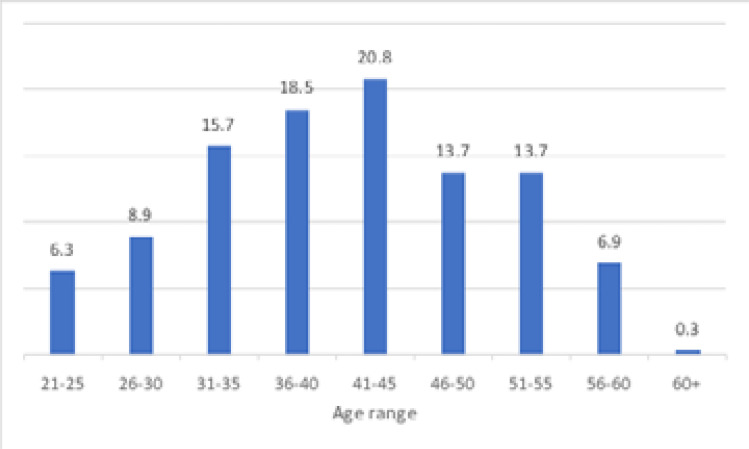
Age Distribution

With regards to age, 20.8% of the participants were in the age group 41–45 followed by age group 36–40 (18.5%). (Fig 1).

**Figure 2:**
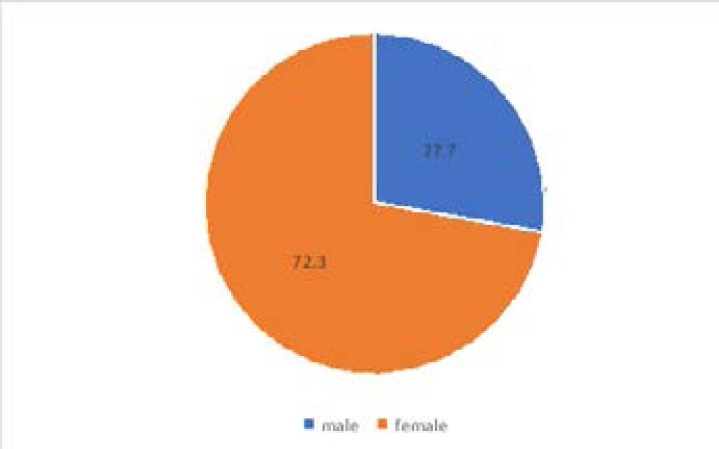
Gender Distribution among participants

Majority (72.3%) of the participants were females as opposed to 27.7% males (fig 2).

**Figure 3:**
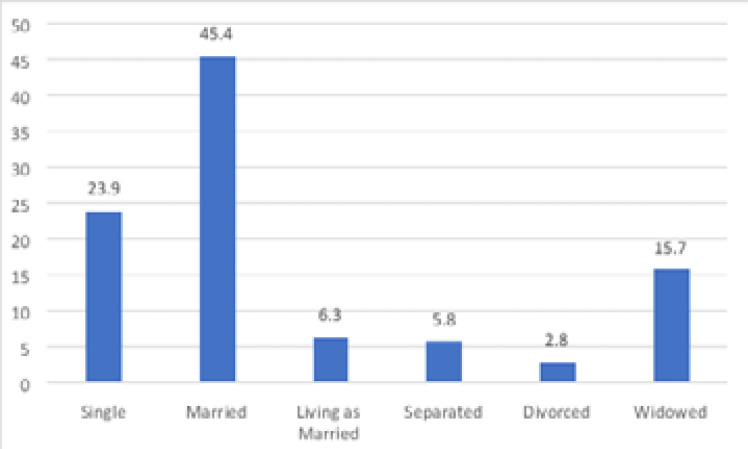
Marital status of participants

A significant proportion of the participants (45.4%) were married while 23.9% were single (fig 3) and most of the participants (44.7%) had primary level education.

**Figure 4:**
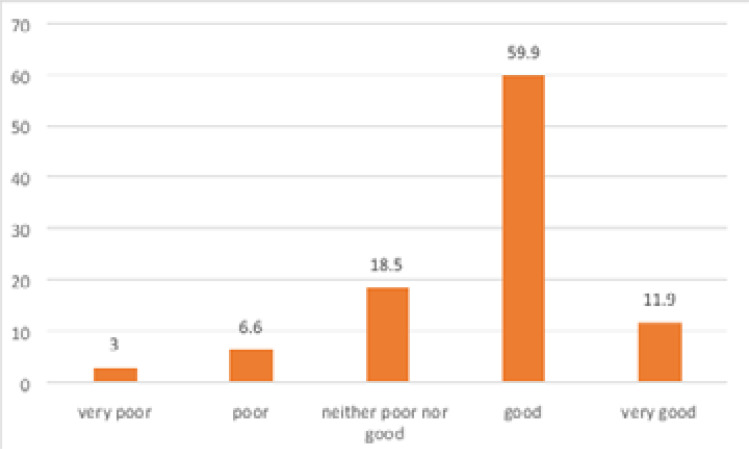
Health status of participants

With regard to their serostatus, 73.4% of the participants were asymptomatic, 19.8% were symptomatic and 6.9% were AIDS converted. Concerning the health status as perceived by the participants, 59.9% admitted being in ‘good’ health while on ART while 18.5% were neutral about their health status (neither in good nor bad health) (fig 4). At the moment of the survey, 33.5% of the respondents felt sick from various ailments ranging from cold, cough, fever, pains on various body parts, headache, body weakness etc

**Figure 5:**
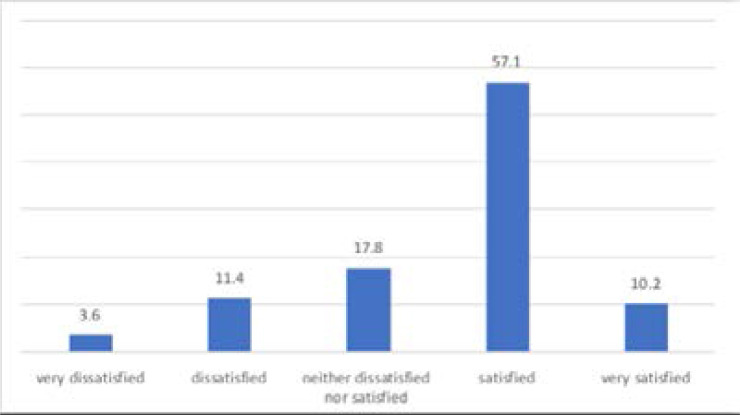
Satisfaction with current health

With regards to their satisfaction on their current health status, majority (57.1%) of the participants were satisfied with their health status and 11.4% were dissatisfied about their current health status (figure 5)

### Bivariate Analyses between QoL and Socio-Demographic Variables

Firstly, bivariate analyses were carried out between QoL and socio-demographic variables and other health status variables. This revealed a significant relationship between QoL and, educational level, HIV serostatus clients' perception of his/her health status, if the client was currently ill during the time of the survey and the client's level of satisfaction with his health status. (Table 1).

**Table 1:** Bivariate Regression Analyses between QoL and Socio-Demographic Variables

Socio-demographic variable	Standardized Coefficient	t- values	p-values
a. Marital status	-0.05	-0.967	0.334
b. Education	0.14	2.868	0.004
c. age range	0.023	0.464	0.643
d. Gender	0.11	0.663	0.508
e. HIV serostatus	-0.36	-7.594	0.0001
f. Health status	0.64	16.509	0.0001
g. currently ill	0.411	8.924	0.0001
h. satisfaction with health	0.639	16.428	0.0001

### Bivariate Analysis between QoL and the Independent Variables

Bivariate analyses conducted between respondents' rating of QoL (dependent variable) and physical, psychological, Level of Independence, environmental, social relations and SRBP domains each as independent variables in their respective models showed a significant association between QoL and the respective independent variables (Table 2)

**Table 2:** Bivariate Analysis between QoL and the Independent Variables

Independent Variable	Standardized Coefficients	t-value	p-value
Physical domain	0.402	8.697	0.0001
psychological domain	0.568	13.676	0.0001
Level of Independence	0.529	12.341	0.0001
Social Relations	0.479	10.796	0.0001
SRPB domain	0.466	10.432	0.0001
Environmental domain	0.485	10.987	0.0001

### Multivariate Regression Analysis between QoL and all Independent Variables

When a multivariable linear regression analysis was done with QoL rating, level of independence was not significantly associated with QoL. This may be explained by its low coefficient (0.019) when compared with the other independent variables.

### Composite Scores for Individual Variables

This reflects individual weightings for each independent variable investigated. Findings showed that spirituality domain had the highest mean score while Environmental domain had the least mean score (Table 4).

**Table 4:** Composite Scores for Individual Variables

Independent Variables	Mean score	Percentage Composite Score
Physical Domain	3.55	71%
Psychological Domain	3.31	71.4%
Level of independence	3.79	75.8%
Social Relationships	3.21	64.2%
Environment Domain	2.82	56.4%
SRPB Domain	3.84	76.8%

### Explaining General QoL of Respondents

The average QoL of the respondents was good with a composite mean score of 3.57 and a percentage composite score of 71.4%

## Discussion

The findings revealed that the overall QoL among the participants was good (71.4%). This result is consistent with similar studies conducted in China. 13. This study contrasts other studies that revealed QoL among people on ART was poor 14, 15. However, this difference can be accounted for by the fact that this current study involved both sexes unlike the former which involved only one sex participant.

HIV serostatus had a negative relationship with OoL with a regression coefficient of -3.6. From the data collection tool, this implies that being asymptomatic was attributed with a higher QoL than being symptomatic and AIDS converted in that order. This finding is in line with studies from Yetnayet AW, Melaku H.L et al 14, 15,17. This could be explained by the fact that the presence of HIV and AIDS symptoms can negatively affect the patient's physical and psychosocial health and thus reduce their overall perception of their QoL.

Another important variable that was found to be strongly associated with QoL was the patients' perception of their current health status. This implies that PLHIV who have accepted their status and consider themselves to be well and living a positive healthy life are more likely to have better QoL scores than those who consider themselves sick because they are on ART. Similarly, it was found that the client's level of satisfaction with their health status was significantly associated with QoL with a regression coefficient of 0.6. This implies that clients who have accepted their status and feel satisfied with various areas of their lives will also perceive higher QoL.

Among the six domains investigated, on a scale of 100, the Spirituality domain was found to have the highest mean score, followed by Level of independence, Physical, Psychological, Social and environmental. This finding concurs with studies from Ma Liping et al which also revealed least scores for environmental and social domains 13. However, the findings differ from many other studies which had social relationship as main predictor of QoL 14, 15. The discrepancy may be attributed to the differences in the study areas, periods, and sociodemographic changes across the study populations.

In terms of the regression coefficients, psychological domain had the highest regression coefficient, followed by physical, environmental, spiritual and then social relationships with QoL. This finding was in line with another study in Karnataka 16 which revealed that psychological factors were the main predictors of QoL. However, the current study was also found to differ with studies carried out in North India and Ethiopia,^14^, ^15^ which revealed that social relationships was the main predictor of QoL among patients on ART. The discrepancy may be attributed to the differences in the study areas, periods and sociodemographic changes across the study populations.

## Conclusion

This study sought to evaluate clients' perception of their physical health, psychological status, social relationships, environmental health, and spiritual/Religion/personal beliefs while on ART. Their perceptions of these various domains determine the perception of their overall QoL. According to clients' perception, they had an average score for their physical health which in this study examined pain, physical problems related to HIV infection, sleep, and energy; physical problems related to HIV infection was the main predictor for physical health. Secondly, they had moderately low perceived scores on their social support and relationships which considered feelings of acceptance, satisfaction with personal relationships, satisfaction with sex life and satisfaction with support from friends; sexual satisfaction was found to be the main predictor for social relationships. Similarly, they had a moderately low perceived score for their psychological health; which assessed domains like enjoyment of life, ability to concentrate, acceptance of bodily appearance, self-satisfaction and feelings of despair, anxiety, depression etc.; acceptance of bodily appearance was the main predictor for psychological health. Most remarkably, there was poor perception on their environmental health which included an assessment on feelings of safety in daily life, healthy physical environment, money to meet daily needs, availability of needed information, opportunity for leisure activities, satisfaction with living conditions, satisfaction with access to health services and satisfaction with transport; opportunity for leisure activities was the main predictor of environmental health. Conversely, clients had highest perception on their SRPB among the studied domains although the score was still somewhere between “neither good nor good and good”, indicating a fairly good perception. Aspects examined under this domain include; meaningfulness of life, being bothered by people's blaming on positive status, fear of the future and worriment about death with worriment about death being the main predictor for Spiritual health. Although much is being done to enhance adherence to care, findings from this study have revealed that clients have low perceptions of other aspects of their health which negatively affect their QoL. Poor QoL has been attributed to poor treatment outcomes, treatment adherence, and retention. Thus, necessary measures should be taken to holistically address all health domains of patients on ART to improve QoL, adherence, and treatment outcomes.

## Limitations

Our study had several limitations. Firstly, the WHO-QOL-BREF instrument measures QoL within two weeks prior to the interview, recall bias may influence the information obtained. Secondly, this study is a cross-sectional design survey, thus, it is difficult to make any causal conclusion between the independent variables and QoL. In future, a prospective study needs to be conducted to confirm the findings of this study.

## Figures and Tables

**Table 3:** Multivariate Regression Analysis between QoL and all independent variables

Independent Variables	Standardized Coefficients (B)	t-value	p-value
Physical domain	0.190	3.789	0.0001
Psychological domain	0.213	2.980	0.003
Social relations domain	0.122	2.133	0.034
Level of independence	0.019	0.267	0.794
Environmental domain	0.147	2.508	0.013
SRPB domain	0.158	3.259	0.001
